# Polyaspartamide Functionalized Catechol-Based Hydrogels Embedded with Silver Nanoparticles for Antimicrobial Properties

**DOI:** 10.3390/polym10111188

**Published:** 2018-10-25

**Authors:** Milène Tan, Youngjin Choi, Jaeyun Kim, Ji-Heung Kim, Katharina M. Fromm

**Affiliations:** 1 Department of Chemistry, University of Fribourg, Chemin du Musée, 9, 1700 Fribourg, Switzerland; Milene.tan@unifr.ch; 2 School of Chemical Engineering, Sungkyunkwan University, Suwon 440-746, Korea; mang9z24@gmail.com (Y.C.); kimjaeyun@skku.edu (J.K.); kimjh@skku.edu (J.-H.K.)

**Keywords:** hydrogel, polyaspartamide, catechol, silver, antimicrobial

## Abstract

In this study, polyaspartamide-based hydrogels were synthesized by boron-catechol coordination followed by incorporation of AgNPs into the materials. Free catechol moieties were exploited to produce AgNPs. TEM analyses displayed AgNPs of less than 20 nm in diameter and with minimum aggregation, attesting the role of hydrogels to act as an efficient template for the production of dispersed particles. XRD analyses confirmed the mean particle size using the Scherrer equation. Release kinetic studies were performed in DMEM medium, showing a slow release over a long time-period. Finally, the MIC and MBC were determined, demonstrating a bacteriostatic and bactericidal effect against Gram-positive *S. aureus* and Gram-negative *E. coli*.

## 1. Introduction

With the increasing use of polymeric biomaterials to replace body parts and functions, device-related infections based on biofilms have led to significant healthcare issues against which antibiotic treatment is complicated, if not useless [1,2]. Therefore, it is important to render the material surfaces antiadhesive or enabling them to kill the adhering microorganisms by incorporating biocidal groups [3,4,5]. Among the different compounds studied so far for this purpose, silver is one of the most promising to focus on, as it is known to exhibit antimicrobial properties even at low concentrations (0.1–10 ppm) [6,7]. It is efficient against a broad range of microorganisms and possesses a low toxicity toward humans [8]. Moreover, silver nanoparticles, AgNPs, are able to release silver ions in the presence of oxygen and humidity over a long time-period [9]. In order to address the issues of aggregation, stabilization and control of the release of silver ions, AgNPs can be incorporated into scaffolds such as hydrogels to form so-called nanocomposites [10,11].

Hydrogels are defined as three-dimensional networks of polymeric chains, able to absorb a certain amount of water (min 10%) without dissolving or losing their 3D structures [12,13]. Due to their high water content, they are highly biocompatible and possess an elasticity comparable to natural tissues [14,15,16]. Moreover, the high mobility of the polymeric chains in the network minimizes the adsorption of proteins and cells at the surface, reducing the risk of thrombosis and infection [17,18,19].

Polyaspartamide-based materials have attracted a significant interest in biomedical applications owing to their biocompatibility, biodegradability and non-antigenicity [20,21]. More importantly, they can be functionalized via aminolysis of polysuccinimide (PSI) with specific functional groups able to bind and immobilize AgNPs in the matrix. The immobilization of the AgNPs is a key feature that needs to be controlled in order to minimize the uptake of the NPs by immune cells, guaranteeing low cytotoxicity [22]. In this context, catechol moieties are interesting as they are able to coordinate metal ions and, above all, to induce the growth of metal nanoparticles without using an additional reducing agent [23,24]. Fullenkamp et al. already reported the synthesis of dopamine (DOPA)-based poly(ethylene glycol) (PEG) hydrogels with AgNPs [25]. They showed a slow silver ion release of only 1% of the total silver amount over two weeks, while still displaying sufficient antimicrobial properties against *S. epidermidis* and *P. aeruginosa*. More recently, Huang et al. described the preparation of catechol-functional chitosan/AgNP composites. These materials demonstrated a high effective bactericidal activity against *E. coli* (MBC 14 µg/mL) and *S. aureus* (MBC 25 µg/mL) [26].

Herein, we report the synthesis of catechol-functionalized polyaspartamide-based hydrogels. These hydrogels were prepared based on the catechol-boron coordination as studied previously [27,28]. The formation of AgNPs is induced by free catechol moieties, without any other reducing reagent. Their release kinetics was studied as well as their antimicrobial activity towards *E. coli* and *S. aureus*.

## 2. Materials and Methods

### 2.1. Materials and Characterization

Boric acid (Merck, Schaffhausen, Switzerland), silver nitrate, NaOH (Aldrich, Buchs, Switzerland), dopamine hydrochloride (DOPA, Aldrich, Buchs, Switzerland), dibutylamine (Alfa Aesar, Karlsruhe, Deutschland) and ethanolamine (EA, Alfa Aesar, Karlsruhe, Deutschland) were used as received.

Nuclear Magnetic Resonance (NMR) spectra were recorded on a Bruker Avance III spectrometer at 400 MHz in DMSO (PSI) and DMF (poly(AspAm(DOPA/EA)). The microporous structure of the hydrogel was determined by field emission scanning electron microscopy (FE-SEM) using a JEOL, JSM-7500F (the operating voltage = 30 kV). The hydrogels were freeze-dried after swelling and cross-sectioned. The morphology and the size of the AgNPs were determined by transmission electron microscopy (TEM) using a FEI/Philips CM 100 Biotwin transmission electron microscope (the operating voltage = 80 keV, in bright-field mode, FEI, Zürich, Switzerland). The nanocomposite was placed in MeOH and sonicated for 30 min to allow the diffusion of the AgNPs out of the gel. The size and size distribution of the nanoparticles were measured and analysed using a ImageJ software.

UV/Vis spectra were recorded with a UV/Vis Spectrometer (Lambda40, Perkin Elmer, Schwerzenbach, Switzerland) at wavelengths ranging from 250 to 600 nm. The thermogravimetric analysis (TGA) measurements were performed on a TGA/SDTA 851e (Mettler Toledo, Bussigny, Switzerland). The measurements were done under air conditions with a rate of 5 °C/min from 25 °C to 500 °C starting by an isotherm of 30 min at 50 °C. Powder X-ray diffractograms (PXRD) were collected on a Stoe StadiP (STOE, Darmstadt, Germany) using Cu K_α1_ radiation (1.5406 Å). The crystallite sizes were obtained using a Match! software.

### 2.2. The Polysuccinimide (PSI) Synthesis

l-aspartic acid (30 g) and 98% *ortho*-phosphoric acid (30 g) (50:50 wt. ratio) were introduced into a round-bottom flask and mixed at room temperature. The mixture was heated slowly to 180 °C under reduced pressure in about 30 min and then maintained at 180 °C for 4.5 h. The reaction mixture was then cooled down and DMF was added to dissolve the product. The resulting solution was precipitated in excess water and the precipitate was washed several times with water to remove residual phosphoric acid until the solution reached a pH of 7. The product was finally dried at 70 °C under vacuum for three days to yield a white powder. The molecular weight was estimated to be approximately 47,000 Da, as calculated from an empirical equation relating the solution viscosity to the molecular weight [29].

### 2.3. The Poly(AspAm(DOPA/EA)) Synthesis

1 g of PSI was dissolved in 30 mL of DMF in a three-neck round-bottom flask and 1.303 g of DOP (80 mol % based on succinimide unit) and 1.386 mL of n-dibutylamine (C_4_H_11_N) were subsequently added. The flask was placed in an oil bath maintained at 80 °C and the reaction was carried out under a nitrogen atmosphere for 24 h in the presence of 0.05 g of Na_2_SO_4_ to prevent oxidation of dopamine groups. An excess (0.385 mL) of EA was then added slowly to the reaction mixture and the mixture was stirred at room temperature for another 24 h. The resulting mixture was precipitated in 500 mL of cold acetone (2×) and washed three times with water.

### 2.4. Hydrogel Synthesis

50 mg polyAspAm (DOP50/EA) was dissolved in 0.5 mL DMF, into which a sufficient amount of B(OH)_3_ solution (catechol/B(OH)_3_ mole ratio = 2.0) was added. The pH of the mixture was increased to the desired final pH of 9 by the addition of a 1 M NaOH solution under nitrogen. The mixture was mechanically mixed to produce a gel via boron-catechol coordination binding. The gel was then washed for 24 h in milliQ water to extract the soluble fraction. 

### 2.5. Incorporation of AgNPs

20 mg of dried gel was placed in 2 mL of AgNO_3_ solution (8% and 12% of weight percentage) and left for 24 h in the dark. The sample was then freeze-dried to obtain the final nanocomposite.

### 2.6. Silver Loading

The gel was placed into concentrated nitric acid (65%) overnight to dissolve all the AgNPs and to destroy the gel. H_2_O was then added to reach a HNO_3_ concentration of 10%.

The silver loading was determined by ICP-OES with a calibration range between 1 and 10 ppm.

### 2.7. Release Kinetics

20 mg of the gel were placed in 2 mL of Dulbecco’s Modified Eagle Medium (DMEM, high glucose, pyruvate, Gibco, Illkirch Cedex, France) supplemented with 10% foetal bovine serum (FBS) at 37 °C.

At different intervals of time, the medium was removed and replaced with new medium. The experiment was performed in sterile conditions under a laminar hood. The Ag concentration was determined by ICP-OES with a calibration range of 10 ppb–2 ppm. The standards were prepared with the same medium used for the experiment.

### 2.8. Biocompatibility Test

First, the materials (hydrogel with and without silver) at different concentrations (0.1, 1 and 5 mg/mL) were incubated in cell culture medium (DMEM, Dulbecco’s Modified Eagle’s Medium) at 37 °C for 24 h. The culture medium was then collected and filtered to remove any residual materials. In the second step, 5000 of RAW267.4 cells (macrophages) were seeded in 96-well plates and the collected cell culture medium was added and incubated for 24 h. Cells serving as untreated control (cells without materials) were incubated with fresh medium and used as references for 100% cell viability. 

The cell viability was evaluated by the MTT (3-(4,5-dimethylthiazol-2-yl)-2,5-diphenyltetrazolium bromide) assay. The assay is based on the accumulation of dark blue formazan crystals inside living cells after their exposure to MTT (20 µL, 5 mg/mL). After four hours of incubation with MTT, the cell culture medium was removed and replaced with dimethylsulfoxide (DMSO) to permeabilize the cell membrane which resulted in the liberation and solubilisation of the formazan crystals. The concentration of formazan was quantified by measuring the absorbance at 550 nm with a UV/Vis spectrometer.

### 2.9. Antimicrobial Tests: MIC and MBC Determination

Bacterial strains and growth conditions:

*Escherichia coli* (*E. coli*) bacteria (ATCC^®^ 25922) were freshly grown in a Mueller Hinton broth (MHB) (Aldrich, Buchs, Switzerland) overnight at 37 °C with shaking (180 rpm). Bacterial numbers were estimated by determining the turbidity (McFarland) at OD600 [30].

*Staphylococcus aureus* (*S. aureus*) bacteria (SA113 wt) were freshly grown in Tryptic soy broth (TSB) for 6 h at 37 °C with shaking. They were then diluted (1:100) in 10 mL MHB and grown overnight at 37 °C and 150 rpm.

Different amounts of the compounds were placed in MH broth and supplemented with aliquots of a bacterial overnight culture in order to reach 10^4^ CFU/mL.

A bacteria culture without the compound was used as positive control and MH broth without bacteria was used as sterility control. The cultures are incubated at 37 °C with shaking (150 rpm) for 24 h. The aliquot which showed no OD were spread on agar plates to determine the MIC and MBC. The tests were performed in triplicate.

## 3. Results and Discussion

### 3.1. Synthesis of the Hydrogels

**Scheme 1:**
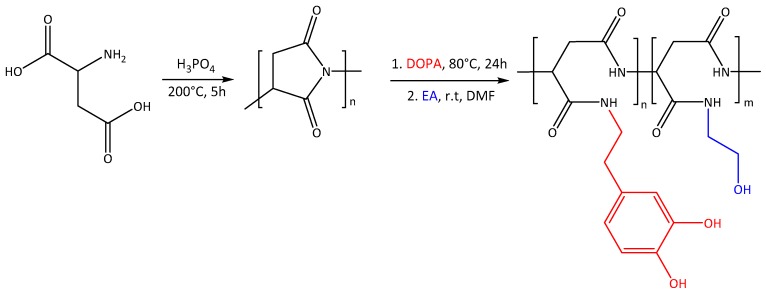
Synthesis of the poly(AspAm(DOPA/EA)).

The polymer poly(AspAm(DOPA/EA)) was obtained by two successive aminolysis reactions of PSI with dopamine hydrochloride and ethanolamine in DMF for 48 h (Scheme 1). 

**Figure 1:**
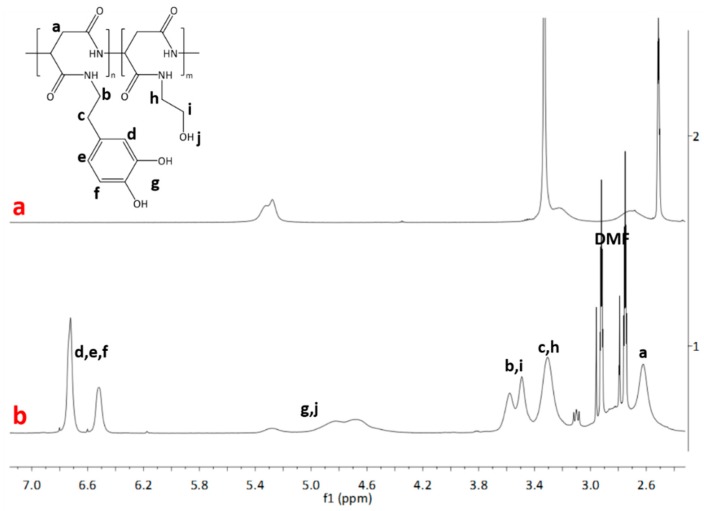
^1^H-NMR spectra of polysuccinimide (PSI) (**a**) and dopamine-conjugated polyAspAm derivative (**b**).

The structure and composition of the PSI and the resultant polymer after aminolysis was confirmed by ^1^H-NMR (Figure 1). The signal for the methine proton (5.28 ppm) of the initial succinimide ring (Figure 1a) disappeared completely (Figure 1b), attesting the successful ring-opening reactions and post-modifications with the desired pendant groups. Figure 1b represents the ^1^H-NMR spectrum of the poly(AspAm(DOPA/EA)) (see Supplementary Information for ^1^H-NMR with integrals in Figure S1) where the peaks **d**, **e** and **f** (6.52–6.72 ppm) can be attributed to the aromatic protons of the dopamine group and peaks **i** (3.49–3.58 ppm) and **h** (3.31 ppm) can be assigned to the methylene protons of the EA, respectively. The relative amounts of each group in the polyAspAm(DOPA/EA) copolymer (content % 39/61) were determined from the integration ratio between peaks **d**, **e**, **f** and **a** (2.6 ppm).

**Figure 2:**
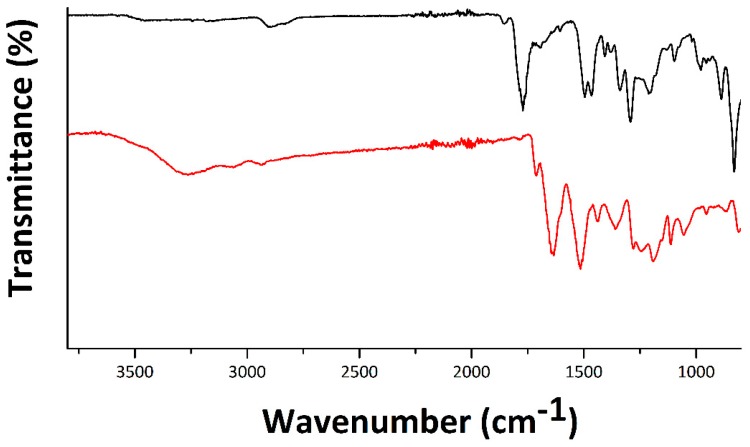
Fourier transform-Infrared (FT-IR) spectra of PSI (black) and Poly(AspAm(DOPA/EA)) (red).

The success of the aminolysis reactions was also confirmed by FT-IR spectroscopy. Figure 2 displays the spectra for PSI (black) and poly(AspAm(DOPA/EA)) (red). The PSI spectrum shows two specific absorption bands around 1700 cm^−1^ and 1380 cm^−1^ which correspond to the stretching of C=O and C–N of the imide ring, respectively. The IR analysis of the copolymer demonstrated disappearance of the characteristic bands of PSI, confirming the ring-opening reaction. The bands at 1644 and 1523 cm^−1^ are attributed to the C=O stretching of the amide groups. Moreover, a broad band in the range of 3500–3200 cm^−1^ attests to the presence of –OH and –NH groups in the copolymer.

**Scheme 2:**
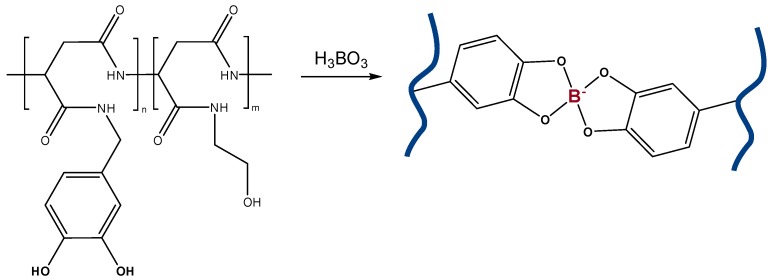
Synthesis of the hydrogel network via boron-catechol coordination.

**Figure 3:**
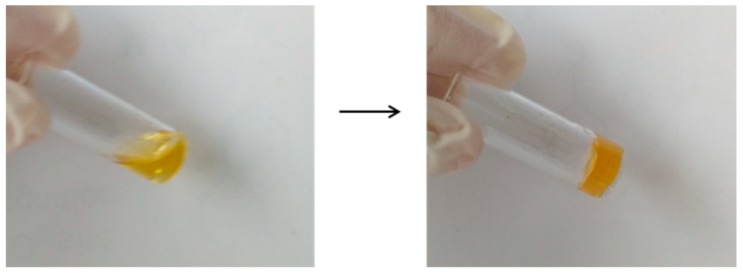
Images of the polymer solution before and after cross-linking.

The polymer was cross-linked via boron-catechol coordination to yield the hydrogel via the formation of four coordinative bonds between the boron and the diols from two catechol groups (Figure 3 and Scheme 2). Vatankhah-Varnoosfaderani et al. previously studied the same type of cross-linking with poly(dopamine methacrylate-co-*N*-isopropylacrylamide) (p(DMA-co-NIPAM)) and obtained multiple stimuli-responsive gels with rapid self-healing [31]. Wang et al. also obtained a polyaspartamide based-hydrogel via the boron-catechol coordination. They showed a reversible gelation depending of the pH with self-healing property [28].

**Figure 4:**
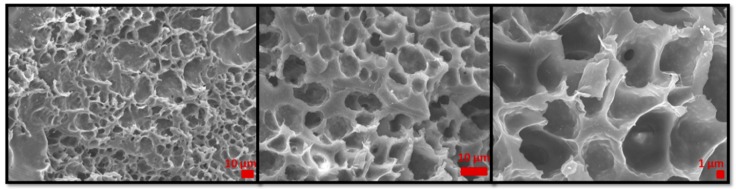
FE-SEM images of the microporous structure of the freeze-dried hydrogels.

The microporous structure of the hydrogels after swelling was determined by field-emission scanning electron microscopy (FE-SEM) (Figure 4). The freeze-dried swollen hydrogel presents a heterogeneous, porous structure at the micrometre scale, with a pore size of less than 15 µm. Wang et al. reported the preparation of the same hydrogels with pore sizes ranging from the submicrometre scale up to several micrometres [27]. As far as we know, no other work based on the same cross-linking showed SEM micrographs of a network structure [31,32].

### 3.2. Preparation of the Hydrogel/Ag Composite and Characterization

**Scheme 3:**
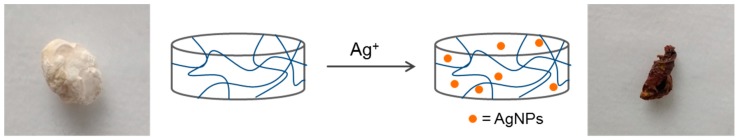
Preparation of the nanocomposite and images of the hydrogel before and after incorporation of the AgNPs.

To provide the antimicrobial properties, silver nanoparticles AgNPs were incorporated into the hydrogels. Practically, the gel was immersed for 24 h in an AgNO_3_ solution at two weight percentages of 5% (sample A) and 7.5% (sample B), allowing the diffusion of silver nitrate solution into the pores, subsequent reduction and dispersion of the AgNPs in the gel network. (Scheme 3) This reduction reaction resulted in a colour change of the gel and of the surrounding medium from transparent/white to dark brown/brown, indicating the production of particles.

**Scheme 4:**
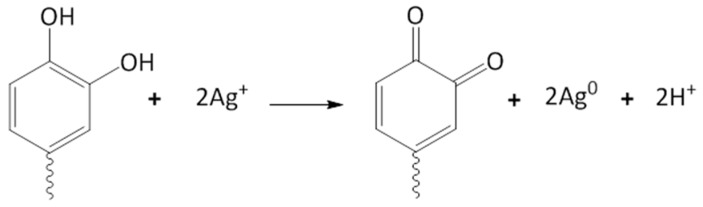
Preparation of the AgNPs with catechol as reducing agent.

The gel was able to reduce the silver ions into AgNPs by exploiting the free catechol moieties contained in the gel, following the reaction described in the Scheme 4. The ability of catechol-based molecules to form metal nanoparticles was already shown by Begum et al. [33].

This method presents the advantages of an alternative green strategy to produce AgNPs without the need for an additional reducing agent and without the formation of possibly toxic by-products [34,35].

**Figure 5:**
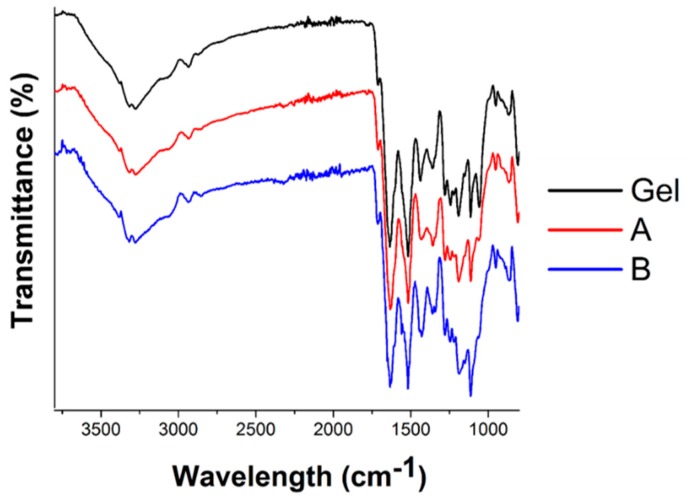
FT-IR spectra of the gel and the nanocomposites.

Figure 5 presents the FT-IR spectra of the gel and the nanocomposites A and B. No significant changes were observed in the presence of AgNPs. The bands at 1632 cm^−1^ and 1527 cm^−1^ correspond to the C=O stretching of the amide groups in the copolymer precursor. The broad band in the range of 3600–2900 cm^−1^ is attributed to the –OH and –NH groups in the copolymer precursor.

The total silver loading was determined by ICP-OES. As displayed in Table 1, the silver loading was low in comparison to the initial amount in the AgNO_3_ solution. To determine the maximum of silver that the gel can incorporate, the gel was immersed into a solution of Ag^+^ (35 wt%) which resulted in a loading of 8% by weight, based on an ICP analysis. The incorporation of silver is highly influenced by the initial concentration of silver ions and increased almost linearly with it. These results indicate that the poor silver content is most likely due to the hydrophobicity of the copolymer precursor, which impedes a high absorption of the AgNO_3_ solution. Indeed, limited solubility in both water and most solvents except DMF was observed for the copolymer, which formed a glue in solution. The poor solubility, as hypothesized by Wang et al. is due to strong intra- and intermolecular hydrogen-bonding interactions between the catechol moieties. This induces aggregation and chain entanglement, thus limiting solubility in water and most solvents [36]. The poor hydrophilicity could be interesting for drug delivery systems as the diffusion of drugs through the network is highly dependent on the water uptake [37]. A low water content affords a slow drug release and it is then possible to maintain a release over few weeks in comparison to a few days for more hydrophilic gels [38].

**Figure 6:**
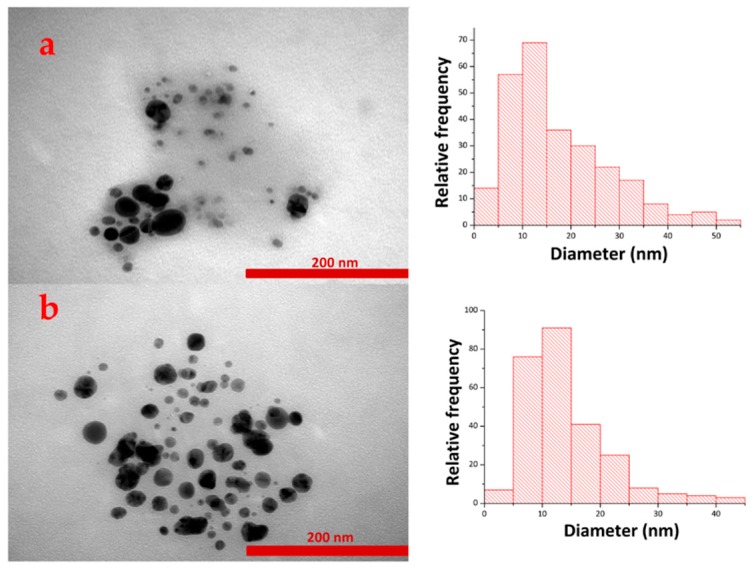
TEM micrographs and size distributions of the AgNPs for the nanocomposite A (**a**) and B (**b**).

**Figure 7:**
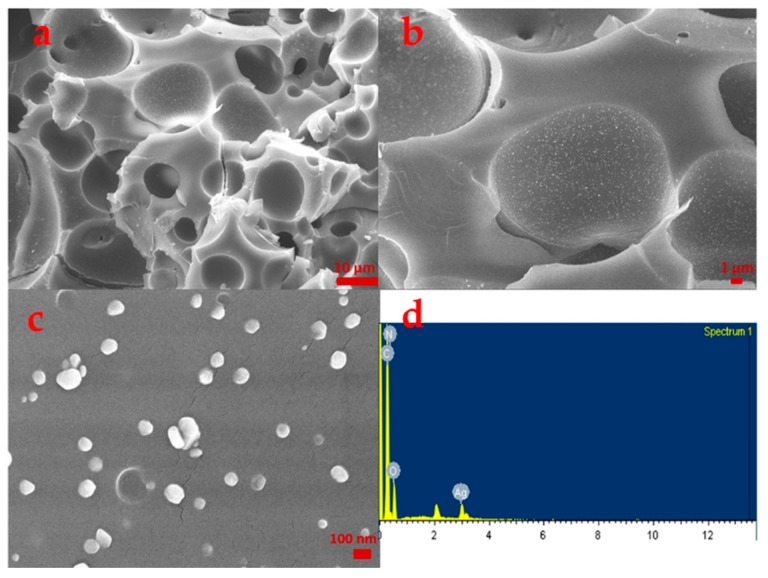
FE-SEM micrographs of the AgNPs in the network (sample B) at three different magnifications (10 µm (**a**), 1 µm (**b**), 100 nm (**c**)) and EDX spectrum (**d**).

TEM micrographs (Figure 6) showed the production of round particles. In both cases, most of the particles displayed a diameter of less than 20 nm. Some larger particles were present but their sizes remained below 100 nm. These results imply that the hydrogels act as an efficient template for the preparation of AgNPs [39,40]. The polymeric chains within the network provide stabilization and easy growth of AgNPs with minimal aggregation, the latter of which is known to reduce the antimicrobial properties [10,41,42]. These results were confirmed by FE-SEM and EDX analyses on the sample B which showed a dispersion and distribution of the particles in the polymeric network (Figure 7).

**Figure 8:**
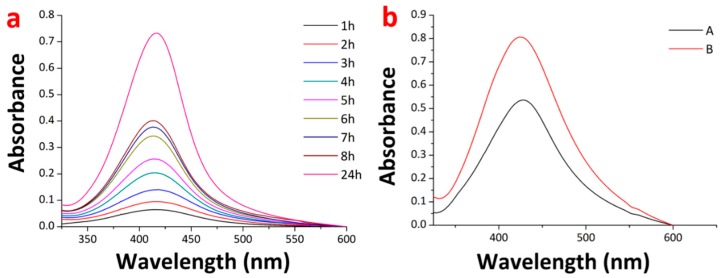
UV/Vis analyses of the surrounding solution over time for sample B (**a**) and after 24 h for samples A and B (**b**).

In order to study and confirm of the formation of AgNPs, UV/Vis measurements were performed on the surrounding solution. As shown in Figure 8a for the sample B, a plasmon band with a maximum absorbance at 418 nm appeared, which is consistent with the formation of AgNPs [43]. Figure 8b presents the UV/Vis analyses for both nanocomposites after 24 h, confirming the presence of AgNPs. The broad band indicates a large size distribution of the AgNPs, in agreement with the TEM analysis. 

**Figure 9:**
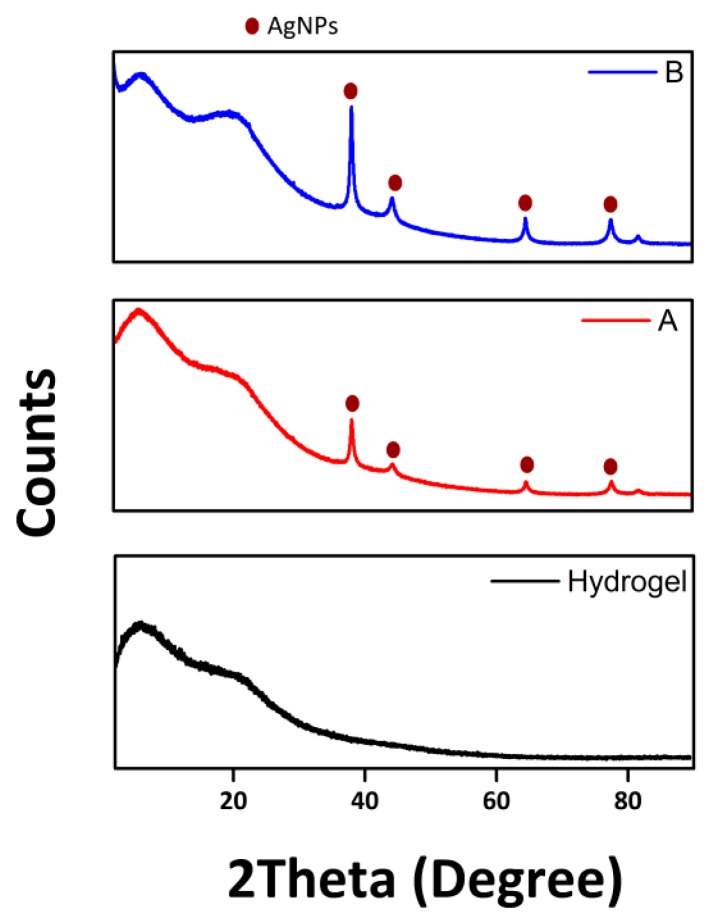
X-ray diffractograms of the hydrogel and the nanocomposites.

X-ray diffraction was also conducted on the nanocomposites (Figure 9). The gel alone displayed an amorphous pattern as expected due to the amorphous nature of the polyaspartamide [44]. In presence of silver, the characteristic peaks at 38.2°, 44.3°, 64.6° and 77.6° corresponding to the (111), (200), (220) and (311) crystal facets of AgNPs were observed [45,46].

From the XRD analyses, the crystallize size of the AgNPs for both samples were calculated using the Scherrer equation (based on (111) as the most intense peak) (Table 2). The AgNPs displayed a crystallite size of approximately 18 nm for both silver contents. These findings are consistent with the results obtained by TEM, where the most common size of AgNPs was around 15 nm in diameter. 

**Figure 10:**
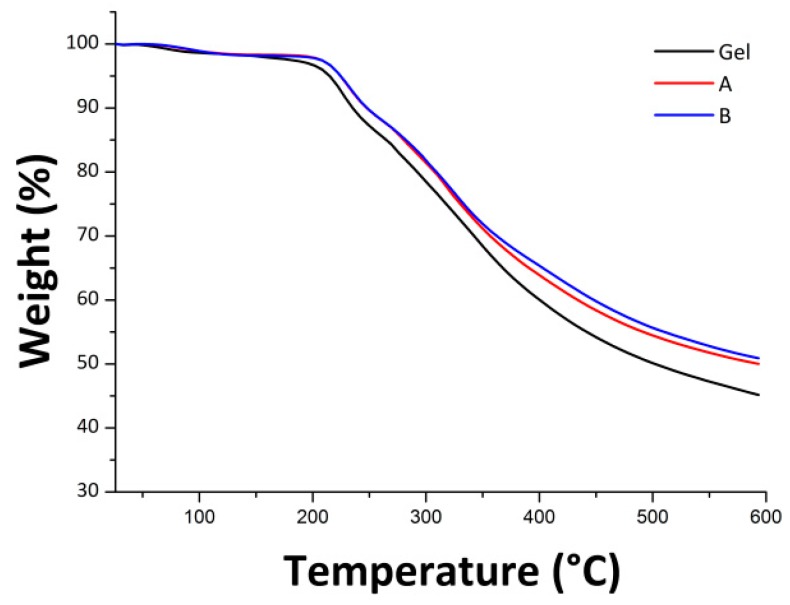
TGA curves of the gel and the resultant nanocomposites.

In order to study the influence of the AgNPs on the thermal stability of the nanocomposites, TGA measurements were conducted in the range from 25 °C to 600 °C. Figure 10 shows a degradation process starting at around 200 °C, which is attributed to the degradation of the polyaspartamide [47]. The gel did not reach 100% of degradation at 600 °C due to the presence of boron which increases the overall stability. Indeed, boron-containing compounds are known to possess flame retardant properties by promoting cross-linking of polymers upon thermal degradation [48]. Moreover, it has also been shown that the presence of hydroxyl groups in the polymer can minimize the decomposition. These OH groups can react with boron to form a coating on the material’s surface which protects it from degradation [49,50].

The nanocomposites exhibited increased thermal stability compared to the hydrogel, with a degradation temperature of 235 °C for the composite and 227 °C for the gel. These findings are consistent with the results obtained by Rao et al. [42]. They showed that Ag-loaded hydrogels had improved the thermal stability in comparison to the initial hydrogels. Furthermore, the difference in weight loss of 4–5% between the hydrogel and the nanocomposites were attributed to the presence of silver. 

### 3.3. Release Kinetics

**Figure 11:**
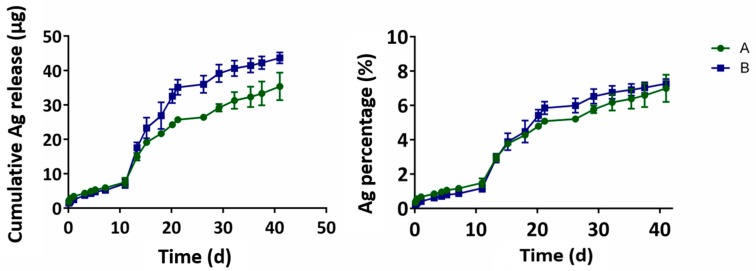
Release kinetics of A and B during 40 days in DMEM medium.

Both nanocomposites were investigated in pellet form for the release of silver ions in the cell culture medium (DMEM + 10% FBS), mimicking the physiological environment. Figure 11a shows the cumulative release of Ag after one month. The total amount released for sample A was 30 µg corresponding to less than 7% of the total amount of Ag inside the material. For sample B, the overall amount released in one month was 40 µg, representing 7.3% of the total amount of Ag contained in the material. For both samples, the release increased by 1.5% after 11 days, which is most likely due to the hydrolysis of the carbonate ester linkages along the polyaspartamide chains [51]. Indeed, degradation of the ester bonds within the network leads to an increase of the internal volume and thus to more water uptake [52]. Giammona et al. already reported the relative stability of the polyaspartamide-based material for up to 10 days with only a partial degradation of the material [53]. Moreover, as the pH increases over time to around 9, the hydrolysis is enhanced due to the formation of carboxylate and alcoholic groups known to accelerate the hydrolysis [54,55]. In addition, the release profile showed a small stabilization around 7.5% of the total Ag amount after 40 days, which followed roughly a first order kinetics with a sustained release of the drug [56]. The slow release profile could be attributed to the high hydrophobicity of the hydrogels leading to poor water uptake and slow diffusion through the network. Consequently, AgNPs have less contact with water and hence release Ag^+^ ions only very slowly. This feature indicates that the materials could be used for long-term applications until the material fully degrades.

### 3.4. Biocompatibility

**Figure 12:**
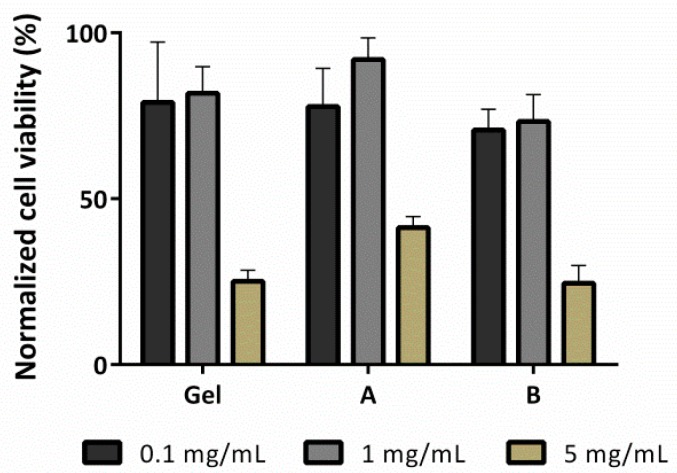
Cell viability of the macrophage cells after treatment with the different materials at different concentrations.

To evaluate the cytotoxicity of these materials by MTT assay, RAW267.4 cells (macrophages) were incubated for 24 h with the cell medium. The cell medium was collected after 24 h of incubation with the different materials (gel, A and B). Regardless of the material types, the cell viability remained over 80% at low concentrations (0.1 and 1 mg/mL) in comparison to untreated control cells (representing 100% cell viability). However, severe cell toxicity (viability lower than 50%) was demonstrated at high concentrations (5 mg/mL) for all the samples (Figure 12). This is in line with previous studies where polyaspartamide had been shown to be biocompatible up to a working concentration of 1 mg/mL [57,58].

The incorporation of silver did not demonstrate higher toxicity than the silver-free material. This observation might be explained by the low silver release at the working concentration which did not induce any cytotoxic effect [59]. Moreover, due to multiple binding sites, AgNPs were possibly immobilized in the matrix, diminishing the cell uptake and cytotoxicity.

### 3.5. Antimicrobial Properties

The antimicrobial activity was evaluated against two clinically relevant bacterial strains, *Staphylococcus aureus* (*S. aureus*) and *Escherichia coli* (*E. coli*). The effect on bacterial growth was investigated to determine the minimum inhibitory concentration (MIC) and minimal bactericidal concentration (MBC). MIC was taken at the lowest amount which inhibits the bacterial growth after 24 h of incubation with an initial loading of bacteria of 10^4^ CFU/mL. MBC was the minimum amount required which showed no bacterial growth on agar plates after 24 h of incubation.

As shown in Table 3, the compounds possessed an antimicrobial effect on *E. coli* and *S. aureus* in the range of 2–5 mg/mL. The performance of the materials could be related to the silver loading and release of silver ions. As the sample B released more silver, the antimicrobial activity was enhanced. In addition, more compound was needed to exhibit a bacteriostatic or bactericidal effect for *S. aureus*. This behaviour can be explained by structural differences between the bacteria. *S. aureus*, Gram-positive bacteria, possess a thick cell wall composed of multiple layers of peptidoglycan compared to Gram-negative bacteria such as *E. coli* which exhibit only a thin layer and a double membrane [60]. Consequently, a higher amount was needed to induce structural damages for *S. aureus*. Moreover, due to this thicker layer, Gram-positive bacteria are less susceptible to silver reaching the cytoplasmic membrane [61,62].

## 4. Conclusions

Polyaspartamide based-hydrogels were prepared via boron-catechol coordination. This preparation was followed by incorporation of AgNPs, exploiting the free catechol moieties as reducing agent. The role of catechols was first to achieve cross-linking and second to reduce and entrap silver. Slow release was displayed in DMEM medium with less than 10% of total amount released within 40 days. The small increase after 10 days was due to the degradation of the polymer. Cell viability tests showed toxicity at high concentration (5 mg/mL). Antimicrobial tests were performed against *S. aureus* and *E. coli* to determine the MIC and MBC of the materials. The amount needed is in the range of 2–5 mg/mL with a concentration of bacteria of 10^4^ CFU/mL. Finally, the materials demonstrated high potential as coating materials for non-vivo long-term applications due to the high toxicity at high concentration.

## Figures and Tables

**Table 1:** Summary and Ag loading of the hydrogel and nanocomposites determined by ICP-OES.

Sample	Ag Initial (%)	Ag Incorporated (W%)
Gel	0	0
A	8%	2.53%
B	12%	3.12%

**Table 2:** Crystallite sizes of the AgNPs obtained using the Scherrer equation.

Sample	Crystallite Size (nm)
A	18.75
B	17.97

**Table 3:** MIC and MBC values of the compounds A and B.

Bacterial Strain	A (mg/mL)		B (mg/mL)	
	MIC	MBC	MIC	MBC
***E. coli***	3.42	3.6	2.66	2.78
***S. aureus***	4.32	4.64	4.24	4.44

## Supplementary Materials

The following are available online at http://www.mdpi.com/2073-4360/10/11/1188/s1.
