# Supplementary material for: Polyaspartamide Functionalized Catechol-Based Hydrogels Embedded with Silver Nanoparticles for Antimicrobial Properties
Source: Polymers (Basel). 2018 Oct 25;10(11):1188. doi: 10.3390/polym10111188 (PMC6290624; doi:10.3390/polym10111188)
Supplement: Supplementary file 1 [file polymers-10-01188-s001.pdf]

Supplementary Information

Polyaspartamide functionalized catechol-based hydrogels embedded with silver nanoparticles for antimicrobial properties

Milene Tan[1], Youngjin Choi[2], Jaeyun Kim[2], Ji-Heung Kim[2], Katharina M. Fromm[1]

[1]University of Fribourg, Department of Chemistry, Chemin du Musée, 9. 1700 Fribourg, Switzerland;
[2]School of Chemical Engineering, Sungkyunkwan University, Suwon 440–746, Republic of Korea

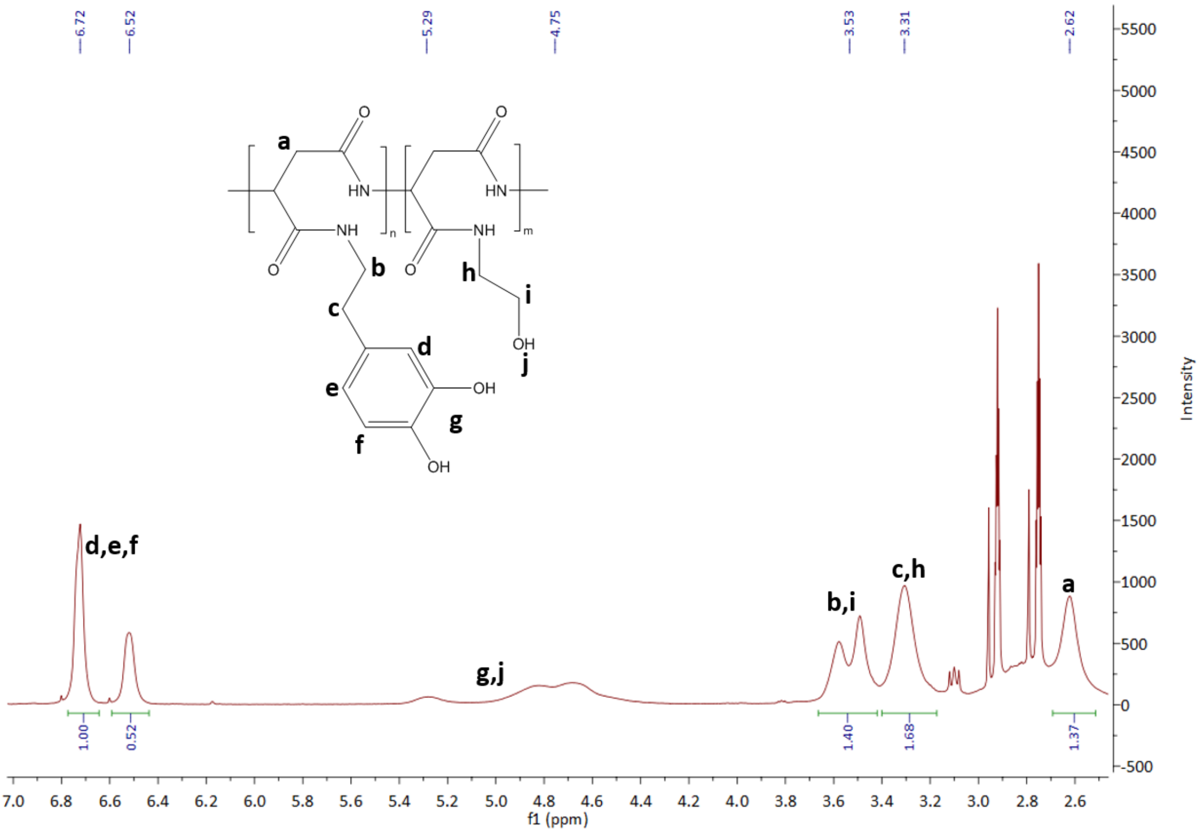

Figure S1 ^{1}H-NMR of the dopamine-conjugated polyAspAm derivative

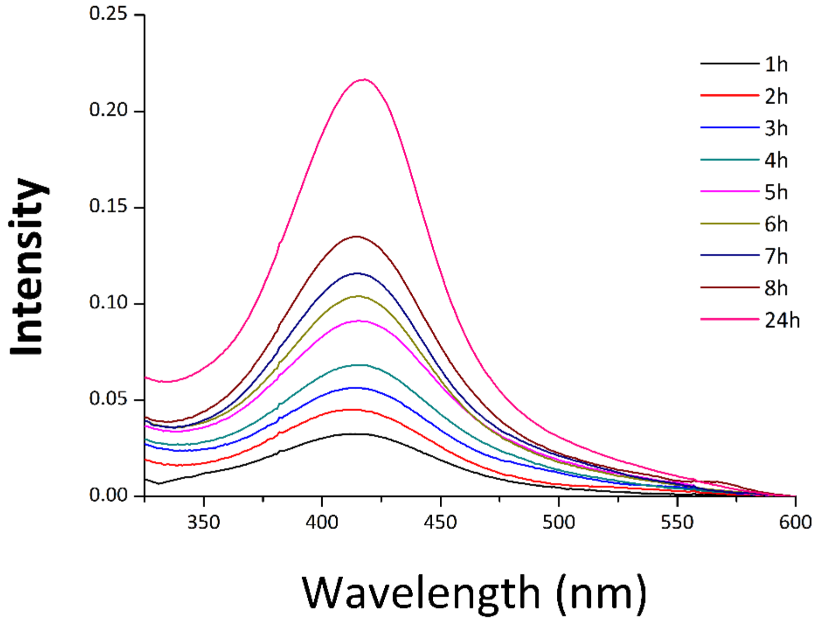

Figure S2 UV-vis analyses of the surrounding solution over time for sample A
